# Research on the prevention of tooth demineralization and the effects and mechanisms of different mineralization solutions on the metabolism of *Streptococcus mutans*

**DOI:** 10.3389/froh.2025.1647945

**Published:** 2025-09-09

**Authors:** Renze Shen, Yongmei Tan, Jinchuan Zheng, Gang Xu, Mingli Lin, Zhanchao Ye, Lingna Han

**Affiliations:** 1 Department of Stomatology, Zhongshan Hospital of Xiamen University, School of Stomatology, Xiamen University, Xiamen, China; 2 Department of Oral and Maxillofacial Surgery, Sun Yat-sen Memorial Hospital of Sun Yat-sen University, Guangzhou, China; 3 Guangdong Provincial Key Laboratory of Malignant Tumor Epigenetics and Gene Regulation, Guangdong-Hong Kong Joint Laboratory for RNA Medicine, Medical Research Center, Sun Yat-sen Memorial Hospital, Sun Yat-sen University, Guangzhou, China; 4 Department of Stomatology, People's Hospital Affiliated of Quanzhou Medical College, Quanzhou, China; 5 Department of Medical Administration, Xiamen Emergency Medical Center, Xiamen, China

**Keywords:** dental caries, prevention, metabolome, acid resistance, glycolysis

## Abstract

**Objective:**

To compare the preventive effects of various mineralization solutions on tooth demineralization and their influence on the metabolism of *Streptococcus mutans (S. mutans)*.

**Methods:**

Pure water, Ca/P mineralization solution, Ca/P mineralization solution with fluoride, Ca/P mineralization solution with zinc, Ca/P mineralization solution with magnesium, and Ca/P mineralization solution with strontium (Sr) were prepared. Tooth fragments were immersed in these solutions at 37°C for 24 h. Surface morphology was examined by scanning electron microscopy (SEM). The relative proportions of surface elements were analyzed, and new substances formed on the tooth surface were identified. Acid-etching was performed to evaluate changes in anti-demineralization ability and wear resistance. *S. mutans* was inoculated onto tooth surfaces, bacterial adhesion was observed using SEM, and water contact angles were measured. Changes in pH and metabolites of bacterial culture media were assessed. KEGG enrichment pathway analysis was conducted to explore metabolic pathways. Amino acids and organic acids in metabolites and bacterial proliferation were evaluated. RT-PCR was used to measure key glycolysis-related gene expression to verify the production of acidic metabolites.

**Results:**

New substances were observed adhering to tooth surfaces by SEM; surfaces treated with zinc and Sr solutions were the roughest. Elemental proportion analysis indicated zinc had the highest adhesion potential, while Sr had the lowest. Newly formed substances included fluorapatite, magnesium hydroxide, and phosphate complexes. All experimental groups demonstrated improved acid resistance and good wear resistance. Sr treatment rendered tooth surfaces more hydrophilic and increased bacterial adhesion. All experimental groups inhibited acid production by *S. mutans*, particularly the fluoride group. Antibacterial tests indicated fluoride and zinc had the strongest antibacterial effects. KEGG pathway analysis suggested that the primary signaling pathways influenced by these substances were related to bacterial antibiotic formation and acid-salt metabolism. Metabolite analysis showed that experimental groups significantly inhibited the formation of acidic amino acids and organic acids, with fluoride exhibiting the most notable effect. RT-PCR results indicated experimental groups suppressed transcription of the glycolysis-related bacterial gene *ldh*, most notably fluoride. Additionally, transcription of bacterial adhesion genes decreased across experimental groups, with Sr markedly inhibiting *spaP* expression.

## 1 Introduction

Dental caries is a significant disease threatening oral health. They cannot heal spontaneously and require treatment for control. Without timely intervention, dental caries may progress to pulpitis or even tooth extraction. The treatment cost and duration gradually increase from caries prevention to dental filling, root canal therapy, and eventually tooth extraction. Proper prevention significantly reduces the incidence of dental caries.

Oral microorganisms play a crucial role in the occurrence and progression of dental caries. Among these, *S. mutans* is considered the most significant cariogenic bacterium (1). The primary cariogenic property of *S. mutans* is its ability to produce acid (2). *S. mutans* metabolizes sugars into pyruvic acid molecules under anaerobic conditions, simultaneously generating ATP for its energy needs. Pyruvate is then converted into lactic acid by *lactate dehydrogenase (ldh)*, lowering the environmental pH (3, 4). In addition to lactic acid, organic acids such as formic acid, acetic acid, and propionic acid also significantly contribute to pH reduction. When acid accumulates sufficiently and environmental pH falls below 5.5, tooth demineralization begins, eventually leading to caries formation (5).

Adhesion is a crucial prerequisite for *S. mutans*-induced dental caries. *S. mutans* adheres to teeth by binding to specific sites on the tooth surface. This adhesion mechanism may involve electrostatic forces, van der Waals forces, and specific molecular interactions. Following attachment, bacteria produce organic acids through glycolysis, leading to tooth demineralization and inhibition of other bacterial growth (6). Therefore, reducing bacterial adhesion is essential in caries prevention. Currently, commonly used preventive methods include reducing bacterial adhesion, decreasing acid production, and enhancing tooth acid resistance (7, 8). The commonly used caries-preventive agents in clinical practice are mainly various mineral solutions with different components. The most common is fluoride; however, it may cause fluorosis. Zinc, strontium, and magnesium also have caries-preventive effects and are considered safer than fluoride, but they are not yet widely used (9–11). Various mineralization solutions differ in composition and caries prevention mechanisms against *S. mutans*. Hence, it is necessary to explore the roles of these mineralization solutions.

Mineralization solutions containing fluoride, zinc, magnesium, and Sr are believed to improve tooth resistance to dental caries. Previous literature suggests that their preventive effects may be associated with improved acid resistance of teeth, reduced bacterial adhesion, and bacterial inhibition. For instance, zinc exhibits antibacterial properties; magnesium disrupts bacterial biofilm formation; and strontium, in combination with fluoride, enhances the acid resistance of teeth (9–11). We hypothesize that when teeth interact with these ions, distinct compounds form on the tooth surface, potentially preventing dental caries by reducing bacterial adhesion, decreasing bacterial acid production, or increasing acid resistance. Moreover, the mechanisms and preventive effects of these ions differ. However, comparative studies evaluating the differences among these substances simultaneously have not been reported. This study investigates interactions between these substances and teeth, identifies newly formed compounds, examines tooth resistance to acid, evaluates their effects on bacterial adhesion, proliferation, and metabolite production such as organic acids. Verification of this hypothesis suggests that mineralization solution, fluoride, zinc, magnesium, and strontium exert distinct effects and mechanisms in preventing tooth decay. Clinically, appropriate agents can be selected according to individual needs.

## 2 Materials and methods

### 2.1 Materials

Zinc chloride (Fisher Scientific, USA); (Fisher Scientific, magnesium chloride (Fisher Scientific, USA), Sr chloride (Fisher Scientific, USA), ultra-pure water system (Chengdu Ultra-Pure Technology, China), Fourier transform infrared spectrometer (Thermo Scientific), x-ray diffractometer (Rigaku), pH meter (MP220, Shanghai Mettler Toledo Instrument Co., Ltd.); PrimeScriptTMRT reagent Kit with gDNA Eraser(Takara, Japan).

### 2.2 Methods

#### 2.2.1 Sample preparation and characterization

Collection of teeth and patient information: A total of 246 volunteers (118 males and 128 females, aged 18–24 years, mean age 20.2 years) with 684 teeth participated in this study. All collected teeth were permanent premolars or third molars extracted for orthodontic purposes. Extractions were performed for clinical treatment needs, not for research purposes. Volunteers were informed about the study's objectives, the intended use of extracted teeth, and relevant procedures. Each volunteer provided informed consent. Teeth were selected based on the following criteria: no caries, no periodontitis, and no use of antibacterial drugs or mouthwash affecting mineralization within the previous six months (Table 1).

**Table 1:** Collection of teeth and patient information.

Groups	Male/female	Age	Number	Premolar/third molar
NC	19/22	19.6 ± 1.4	114	10/104
Ca/P	21/22	19.2 ± 1.2	114	9/105
Ca/P + NaF	23/20	20.4 ± 1.2	114	12/102
Ca/P + Zn	18/21	20.1 ± 0.9	114	11/103
Ca/P + Mg	17/23	20.3 ± 0.7	114	13/101
Ca/P + Sr	20/20	19.1 ± 1.1	114	10/104
*X*^2^/F	1.19	29.56		1.105
*P*	>0.05	>0.05	>0.05	>0.05

#### 2.2.2 Sample processing method

The surfaces of all collected teeth were cleaned using an ultrasonic scaler. All teeth were then subjected to uniform high-temperature and high-pressure sterilization, followed by drying for subsequent use. Teeth were randomly numbered and divided into 6 groups using the random number table method, with allocation performed in a blinded manner. Each group contained an equal number of teeth, and samples were further numbered twice. Zinc chloride, sodium fluoride, magnesium chloride, and strontium chloride were dissolved in the mineralization solution at 37°C to prepare saturated solutions. When these substances were added to pure water, they exhibited either endothermic or exothermic behavior. The solution was deemed to have reached a saturated concentration when its temperature stabilized at 37°C and crystal precipitation was observed. The concentrations of the respective saturated solutions were as follows: sodium fluoride, strontium chloride, magnesium chloride, and zinc chloride at approximately 3.9 wt/vol %, 80.8 wt/vol %, 40.5 wt/vol %, and 162.2 wt/vol %, respectively. The saturated solutions were filtered through a 0.22 μm filter. The experimental groups were as follows: NC group (pure water), Ca/P group (mineralization solution), Ca/P + NaF group (Ca/P + NaF saturated solution), Ca/P + Zn group (Ca/P + Zn saturated solution), Ca/P + Mg group (Ca/P + Mg saturated solution), and Ca/P + Sr group (Ca/P + Sr saturated solution). Teeth were immersed in the designated solutions at 37°C for 24 h, then removed, dried, and stored. All the above operations were conducted in a clean bench. All subsequent experiments used these processed samples. Based on the second numbering, teeth were again randomly assigned to experiments using the random number table and blinded allocation (Table 2). RT-PCR, pH measurements, and bacterial proliferation assays were performed using bacterial culture medium, with all teeth assigned a recorded number of 0. No tooth was reused.

**Table 2:** Sample numbers for various characterization and testing methods.

Testing methods	SEM	XRD, XPS, FTIR	Acid-etched/Non-acid- etched	Abrasion resistance	Water contact angle	Metabolites/RT-PCR/PH/Proliferation	Bacterial adhesion SEM
Number	6	6	3/3	12	3	6/0/0/0	3
Replicates	3	3	3/3	1	3	5	3
Total	18	18	18	12	9	30	9

#### 2.2.3 Characterization of observations and compound composition analysis

For each group, 6 teeth were selected. The surfaces were rinsed with deionized water, dried, and ground into fragments. Surface morphology was examined using a scanning electron microscope (SEM). Energy -dispersive spectroscopy (EDS) was performed to randomly determine the relative content of various elements on 3 surfaces of each tooth. Another 6 teeth from each group were pulverized, and x-ray diffraction (XRD), x-ray photoelectron spectroscopy (XPS), and infrared spectroscopy were used to analyze the possible formation of surface compounds. Three random sites in each powder sample were selected for analysis.

#### 2.2.4 Assessment of tooth acid and abrasion resistance

Changes in acid and abrasion resistance: Three samples from each group were treated with phosphoric acid for 10 s and rinsed with deionized water. The surfaces were observed under a root canal microscope and SEM, and three random fields from each tooth were photographed for comparison. Twelve teeth were subjected to 5 min of friction at 12 N, and surface wear was evaluated by plotting friction depth against time. Three additional samples from each group were tested using a water contact angle analyzer by depositing 2 μl of pure water and recording contact behavior within 5 s.

#### 2.2.5 Evaluation of bacterial adhesion and acid production

Saliva was collected from healthy patients undergoing orthodontic treatment. Samples were obtained either in the early morning with an empty stomach or 2 h after a meal. Prior to orthodontic treatment, all patients received ultrasonic dental scaling, had no history of antibacterial agent administration, and performed mouth rinsing with normal saline as a routine procedure. Patients were asked to sit quietly, and a sterile centrifuge tube was placed under their lower lip to collect naturally flowing saliva. The collection duration was 5 min, and the collected saliva was stored at −80°C. The glycerol stock solution of *S. mutans* ATCC25175 was retrieved from a −80°C refrigerator and thawed on ice. In a clean bench, 10 μl of the thawed bacterial solution was pipetted into 5 ml of brain heart infusion (BHI) liquid medium. The medium was then incubated in an anaerobic chamber at 37°C for 24 h, followed by identification using Gram staining. According to the above method, 6 teeth were randomly selected, including those with bacterial adhesion. The adhesion experiment was repeated 3 times, and the metabolic assay was performed 5 times, involving a total of 6 to 7 teeth. Teeth were then rinsed with deionized water, sterilized by autoclaving, and dried for subsequent analysis. Human saliva was collected and filtered through a 0.22 μm filter. Thirty sterilized teeth from each group were immersed in filtered saliva for 30 min. Nutrient broth containing 1% sucrose was then added to cover the surfaces, and teeth were co-cultured with *S. mutans* for 24 h. All operations were performed in a clean bench, and the co-culture was incubated in an anaerobic chamber at 37°C. After incubation, the teeth were removed, and bacterial adhesion was observed using SEM. Culture media were collected to dynamically monitor pH changes. The optical density (OD) of the culture medium was measured using a microplate reader to assess antibacterial and proliferative effects. The adhesion experiment was repeated 3 times, and the metabolic assay 5 times.

#### 2.2.6 Analysis of acid production and adhesion mechanisms of *S. mutans*

Metabolites and bacteria were collected by scraping the tooth surfaces for metabolomics analysis, which was performed by Majorbio Company. Differences in metabolite composition among the groups were analyzed. KEGG enrichment analysis was conducted to identify potential signaling pathways associated with metabolite changes. Variations in acidic amino acids and organic acids were detected. The roles of key glycolytic signaling molecules in acid production and adhesion of *S. mutans* were verified by RT-PCR.

RT-PCR Procedure: RNA extraction and DNA removal: Bacterial culture solution was collected into a 2 ml centrifuge tube and centrifuged at 4,000 × g for 5 min, followed by a 2 min rest. The supernatant was discarded, and the pellet was resuspended in 1% DEPC-treated water. After discarding the supernatant again, 200 μl of lysozyme was added, and the suspension was incubated at 37°C in a water bath for 4 h. One milliliter of Trizol reagent was added, and RNA was extracted following the manufacturer's instructions for the Takara 9,108 Trizol RNA Extraction Kit. RNA purity was assessed using a UV spectrophotometer and gel electrophoresis. DNA removal was performed according to the manufacturer's protocol for the Takara PrimeScript™ RT Reagent Kit with gDNA Eraser under the following conditions: 42°C, 2 min; 4°C, ∞. Reverse transcription was performed per the same manufacturer's instructions under the following conditions: 37°C, 15 min; 85°C, 5 s; 4°C, ∞. The resulting cDNA was stored at −20°C. RT-PCR was carried out according to the manufacturer's protocol with the following conditions: 95°C, 30 s; 95°C, 5 s; 60°C, 30 s for 1 cycle, for 40 cycles. Amplification and melting curves were confirmed upon completion. Gene expression was normalized to *16S rRNA* using the CT values (Table 3), and relative expression levels of target genes were calculated using the 2^−ΔΔCt^ method.

**Table 3:** Primer sequences for RT-PCR.

Gene	Forward	Reverse	Primer length
*ldh*	TTGGTGATGGTGCTGTAGGTTC	AGCATCCGCACAGTCTTCATA	153 bp
*spaP*	AAGGCTTCTGCTGTGGATGAT	GTGAGTTATTGCTTACTGTCGTTGA	190 bp
*gbpB*	AGCAACAGAAGCACAACCATCAG	CCACCATTACCCCAGTAGTTTCC	150 bp
*16S rRNA*	GCGACGATACATAGCCGACCT	TCCATTGCCGAAGATTCCCTA	103 bp

#### 2.2.7 Data analysis was conducted using SPSS 26.0 software

Measurement data following a normal distribution were expressed as mean ± standard deviation (x ± s), and intergroup comparisons were conducted using one-way analysis of variance. *post hoc* pairwise comparisons were performed using the LSD-t test. Count data were presented as cases (%), and intergroup comparisons were made using the chi-square test or Fisher's exact test. For multiple comparisons, significance was adjusted using the Bonferroni correction, with α set at 0.05.

## 3 Results

**Figure 1:**
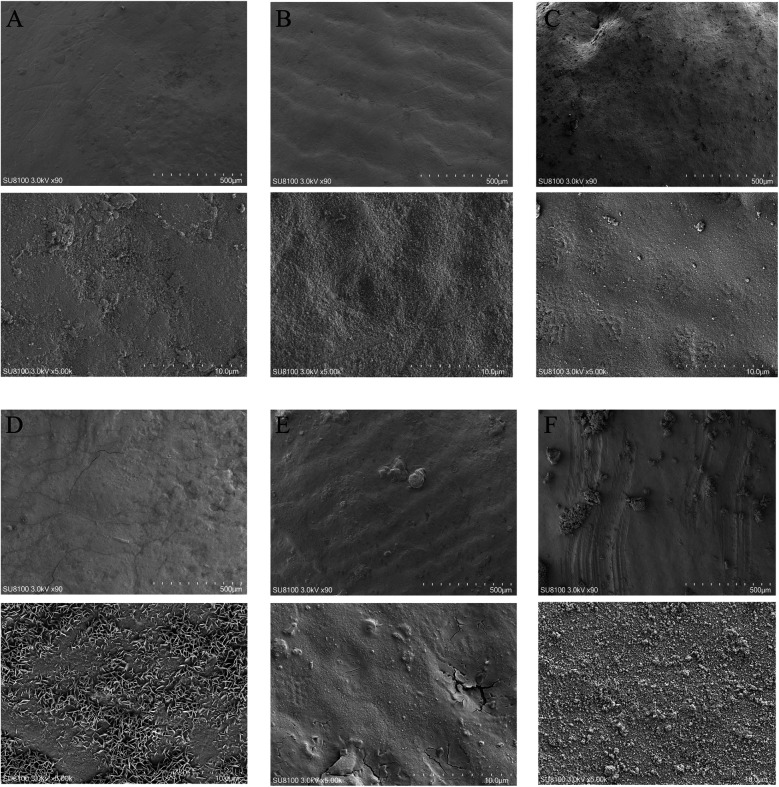
Scanning electron microscope images: **(A)** NC; **(B)** Ca/P; **(C)** Ca/P + F; **(D)** Ca/P + Zn; **(E)** Ca/P + Mg; **(F)** Ca/P + Sr (scale bars: 500 μm, 10 μm).

No statistically significant differences were observed among the tooth groups, indicating good consistency (Table 1). Figure 1 illustrates the surface morphology of teeth after treatment with different ions in a calcium-phosphorus saturated solution. The surface morphology of the Ca/P and Ca/P + F groups was most similar to natural teeth. Particles of 262 ± 54 nm formed on the surface of the Ca/P + Zn group. The surface of the Ca/P + Mg group appeared smooth. Particles of 326 ± 63 nm formed on the surface of the Ca/P + Sr group. The Ca/P group and the Ca/P + F group exhibited the least alterations in tooth surface structure.

Table 4 presents the relative contents of surface elements among the different groups. Under natural conditions, the tooth surface mainly consists of calcium and phosphorus. The contents of fluorine, zinc, and magnesium are very low, and Sr is undetectable. Among these elements, zinc most readily deposits on tooth surfaces. Strontium deposition was second only to zinc, whereas magnesium and fluorine showed the lowest deposition levels. All differences compared with the control group were statistically significant.

**Table 4:** Relative contents of each element.

Elements Groups	Ca (wt%)	*P* (wt%)	F (wt%)	Zn (wt%)	Mg (wt%)	Sr (wt%)
NC	20.5 ± 5.3	10.0 ± 3.5	0.04 ± 0.02	0.14 ± 0.05	0.02 ± 0.10	—
Ca/P	23.4 ± 27.5	12.87 ± 4.4^a^	0.03 ± 0.01	0.10 ± 0.35	0.04 ± 0.01	—
Ca/P + NaF	27.06 ± 6.2	11.52 ± 4.7	1.20 ± 0.53^a^	0.16 ± 0.03	0.06 ± 0.02	—
Ca/P + Zn	13.60 ± 8.5	6.12 ± 5.7^a^^,^^b^^,^^c^	0.06 ± 0.02^c^	15.38 ± 4.38^a^^,^^b^^,^^c^	0.05 ± 0.03	—
Ca/P + Mg	24.22 ± 7.4	9.46 ± 6.1^b^^,^^d^	0.04 ± 0.12^c^	0.13 ± 0.37^d^	1.26 ± 0.05^a^^,^^b^^,^^c^^,^^d^	—
Ca/P + Sr	31.09 ± 8.6^a^^,^^d^	11.87 ± 5.9^d^	0.34 ± 0.22^a^^,^^b^^,^^c^^,^^d^	0.15 ± 0.28^d^	0.08 ± 0.03^e^	5.27 ± 1.26
**F**	3.94	15.69	22.49	68.31	586.60	
**P**	<0.01	<0.001	<0.001	<0.001	<0.001	

a Indicates *P* < 0.05 compared with the NC group.

b Indicates *P* < 0.05 compared with the Ca/P group.

c Indicates *P* < 0.05 compared with the Ca/P + NaF group.

d Indicates *P* < 0.05 compared with the Ca/P + Zn group.

e Indicates *P* < 0.05 compared with the Ca/P + Mg group.

**Figure 2:**
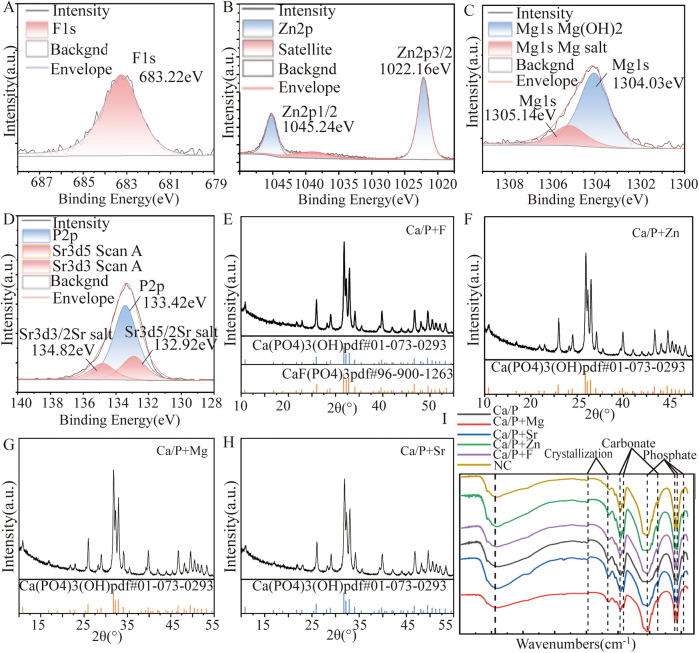
XRD and XPS patterns: **(A,E)** Ca/P + F; **(B,F)** Ca/P + Zn; **(C,G)** Ca/P + Mg; **(D,H)** Ca/P + Sr; **(I)** Infrared diffraction.

Figure 2A shows the XPS spectrum. The spin-orbit peak of fluorine (F) at 683.27 eV is characteristic of metal fluorides, suggesting a reaction between fluorine and hydroxyapatite. In the XRD pattern (Figure 2E), hydroxyapatite and fluorapatite phases were identified, indicating that fluorine had doped into hydroxyapatite to form fluorapatite.

Figure 2B indicates XPS characteristic peaks for zinc (Zn) at 1022.16 eV and 1045.26 eV, corresponding to Zn2p 3/2 and Zn2p 1/2 orbitals, respectively. These peaks match those of zinc metal salts, supporting the possibility of Zn doping into hydroxyapatite. Figure 2F shows only the characteristic peaks of hydroxyapatite. Attempts to match the XRD patterns to calcium phosphate doped with Zn were unsuccessful, possibly due to low content or the formation of amorphous substances. Therefore, XRD results cannot confirm new substances from Zn doping.

Figure 2C reveals two peaks in the Mg1s orbital. The red peak at 1305.1 eV corresponds to Mg(OH)_2_, and the blue peak at 1304.0 eV corresponds to magnesium salts. This suggests Mg may replace calcium and dope into hydroxyapatite. Figure 2H XRD analysis identified only hydroxyapatite peaks. Attempts to match XRD patterns with calcium phosphate doped with Mg also showed poor results. Thus, the XRD data cannot confirm that Mg doping generates new substances in hydroxyapatite.

Figure 2D illustrates the XPS characteristic peaks of Sr at 132.92 eV and 134.82 eV, corresponding to oxygen-containing acid salts of Sr. It can serve as evidence of Sr metal doping into hydroxyapatite. These findings support the possibility of Sr doping into hydroxyapatite, although additional analyses are needed for definitive confirmation. In Figure 2H, only hydroxyapatite characteristic peaks were identified. Attempts to match the XRD patterns to calcium phosphate doped with Sr showed low agreement, indicating that XRD analysis alone cannot confirm the formation of new substances resulting from Sr doping into hydroxyapatite.

Fluorapatite and magnesium hydroxide were the only definitively identified substances formed after the reactions. XRD and XPS results suggested zinc, magnesium, and Sr could react with sulfates, carbonates, and phosphates. Infrared diffraction analysis further confirmed the presence of phosphate and carbonate ions. These findings imply that zinc, magnesium, and Sr may replace calcium ions and react with phosphate ions in hydroxyapatite, and possibly react with carbonate ions from enamel proteins. Additional experiments are required to determine the precise molecular formulas.

**Figure 3:**
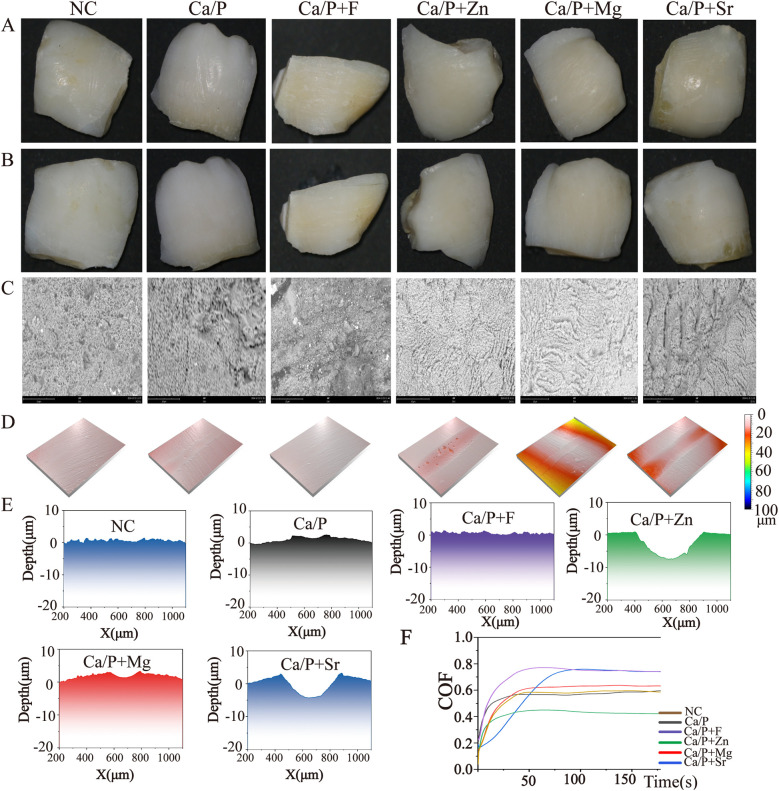
**(A)** Enamel without acid etching; **(B)** acid-etched enamel surface; **(C)** SEM image of acid-etched enamel; **(D)** three-dimensional surface view after friction; **(E–J)** surface depth maps after friction; **(K)** friction depth-time relationship graph.

Results from Figures 3A–C demonstrate that fluorine, zinc, magnesium, and Sr improve acid resistance of the enamel surface, thereby reducing acid etching and demineralization to some extent. Fluorine demonstrated the greatest effect in improving tooth demineralization, followed by zinc, strontium, magnesium, and Ca/P in descending order. Figures 3D,E,K indicate reduced wear resistance for groups treated with fluorine, zinc, magnesium, and Sr, especially for zinc, magnesium, and Sr groups. This suggests fluorine exhibits stronger surface adhesion than the other elements. Zinc exhibited the poorest wear resistance.

**Figure 4:**
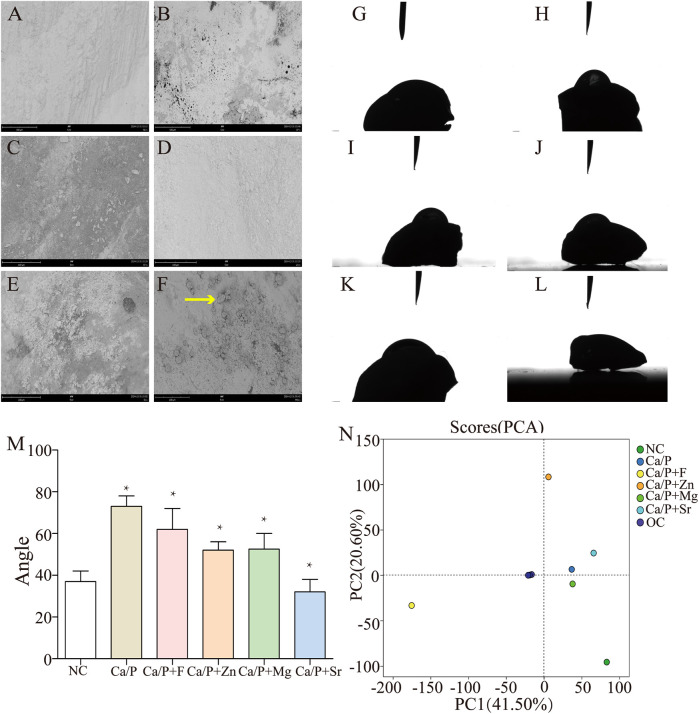
**(A–F)** SEM images after co-culture with *S. mutans* (NC; Ca/P; Ca/P + F; Ca/P + Zn; Ca/P + Mg; Ca/P + Sr); **(G–L)**. Water contact angle diagrams (NC; Ca/P; Ca/P + F; Ca/P + Zn; Ca/P + Mg; Ca/P + Sr); **(M)** Contact angle comparison; N. Metabolite composition diagram.

Figures 4A–F illustrate that after co-culture with *S. mutans*, bacterial metabolites were the primary substances adhering to enamel surfaces, with very few bacteria observed. Only the Sr group showed noticeable bacterial adhesion (Figure 4F red arrow). Figures 4G–L indicate that enamel surfaces from the Sr-treated group exhibited greater hydrophilicity, which may explain the increased bacterial adhesion. Figure 4M shows relative quantification of contact angles. Statistically significant differences were found compared with the control group. Metabolites adhered to tooth surfaces were extracted and analyzed by metabolomics, revealing significant differences in the fluoride-treated group compared to other groups (Figure 4N). Changes in culture medium pH were measured, showing that experimental groups had higher pH values than the control group. The fluoride group exhibited the highest pH, followed by the zinc group. Both groups showed statistically significant differences relative to the experimental group. No significant differences were found between the Ca/P and control groups (Table 5). These findings indicate that fluorine and zinc are the most effective agents in reducing the production of acidic metabolic products by bacteria.

**Table 5:** Ph values.

Groups	12 h	24 h	48 h	72 h
NC	4.86 ± 0.18	3.95 ± 0.12	3.35 ± 0.22	3.90 ± 0.13
Ca/P(*)	4.56 ± 0.09	3.86 ± 0.17	3.98 ± 0.12	4.06 ± 0.12
Ca/P + NaF(*)	6.86 ± 0.08^a^^,^^b^	6.92 ± 0.14^a^^,^^b^	6.85 ± 0.15^a^^,^^b^	6.45 ± 0.24^a^^,^^b^
Ca/P + Zn(*)	6.46 ± 0.07^a^^,^^b^	6.12 ± 5.27^a^^,^^b^	6.28 ± 0.12^a^^,^^b^	6.05 ± 0.12^a^^,^^b^
Ca/P + Mg(*)	5.45 ± 0.14^c^^,^^d^	5.05 ± 0.16^a^^,^^b^^,^^c^^,^^d^	5.05 ± 0.08^a^^,^^b^^,^^c^^,^^d^	5.24 ± 0.7^a^^,^^b^^,^^c^^,^^d^
Ca/P + Sr(*)	5.23 ± 0.24^c^^,^^d^	5.35 ± 0.20^a^^,^^b^^,^^c^^,^^d^^,^^e^	5.05 ± 0.20^a^^,^^b^^,^^c^^,^^d^	5.17 ± 0.27^a^^,^^b^^,^^c^^,^^d^
**F**	229.42	10.75	70.83	99.42
**P**	<0.001	<0.001	<0.001	<0.001

a Indicates *P* < 0.05 compared with the NC group.

b Indicates *P* < 0.05 compared with the Ca/P group.

c Indicates *P* < 0.05 compared with the Ca/P + NaF group.

d Indicates *P* < 0.05 compared with the Ca/P + Zn group.

e Indicates *P* < 0.05 compared with the Ca/P + Mg group.

**Figure 5:**
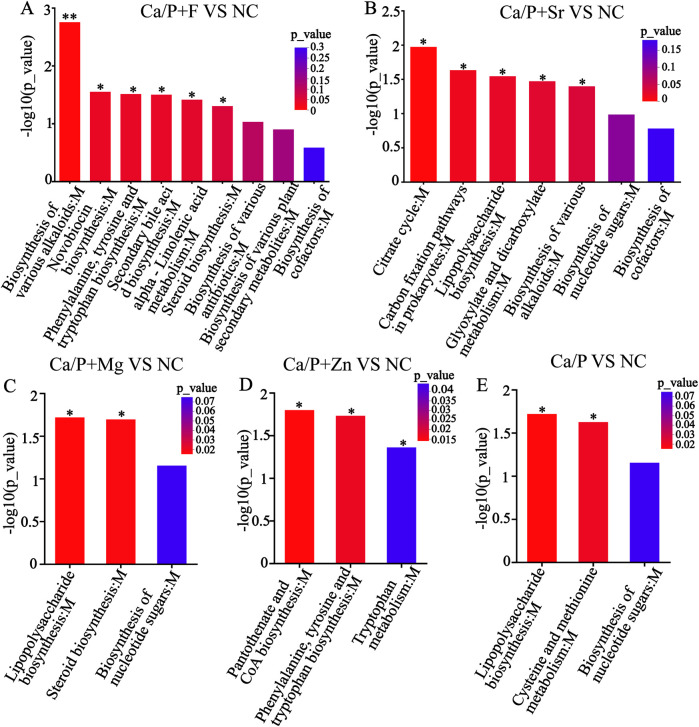
KEGG enrichment analysis **(A)** Ca/P + F; **(B)** Ca/P + Sr; **(C)** Ca/P + Mg; **(D)** Ca/P + Zn; **(E)** Ca/P.

Metabolic pathways were further explored to understand mechanisms underlying bacterial metabolite and pH changes. Figure 5A indicates that compared to the NC group, the fluoride group showed significant pathway differences, particularly in the biosynthesis of various alkaloids, novobiocin, and antibiotics. Figure 5B indicates that the Sr group differed mainly in the citrate (TCA) cycle, carbon fixation pathways in prokaryotes, and alkaloid biosynthesis. Figure 5C shows the magnesium group's differences were primarily in lipopolysaccharide and steroid biosynthesis. Figure 5D indicates that the zinc group mainly differed in pantothenate and CoA biosynthesis, and phenylalanine, tyrosine, and tryptophan biosynthesis. Figure 5E demonstrates that the Ca/P group showed differences primarily in lipopolysaccharide biosynthesis, and cysteine and methionine metabolism compared to the NC group. The BH method was used to correct the *P*-values. When the corrected *P*-value was less than 0.05, it was considered that the pathway had significant enrichment.

**Figure 6:**
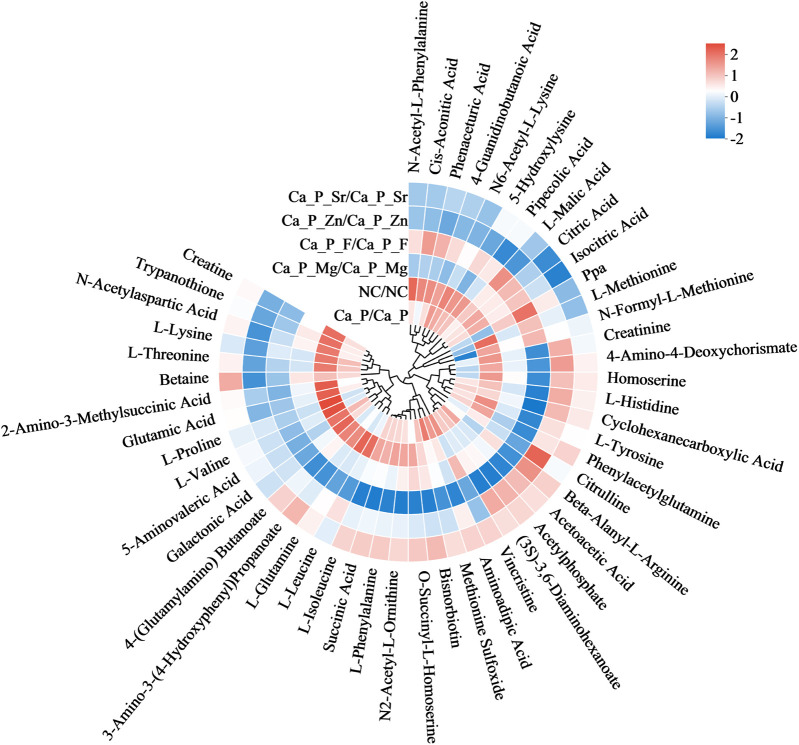
Diagram showing differences in amino acid metabolites.

Based on metabolic pathway analysis, bacterial metabolism and organic acids were further investigated. Figure 6 illustrates differences in amino acid metabolism, where red indicates metabolite enrichment. Compared with the control group, acidic amino acid metabolites in each experimental group decreased. Notably, biological modulators such as creatine, glutamic acid, and galactonic acid showed significant reductions in the Ca/P + F group. The outer circle represents the names of the metabolites. The color indicates the relative abundance of the metabolites in the sample. The tree on the circle represents the clustering of the metabolites, with closer branches indicating a closer similarity in content. Further examination of organic acid metabolites (Table 6) indicated a decreasing trend for organic acids, including lactic acid, across the experimental groups. Additionally, pyruvic acid, a critical glycolysis product of *S.mutans*, and fructose-6-phosphate were also reduced. The differences were statistically significant, suggesting that the glycolytic pathway may underlie the reduced acidic metabolite levels observed in the experimental group compared to the control group.

**Table 6:** Relative contents of acidic metabolites.

Metabolite	NC	Ca_P	Ca_P_F	Ca_P_Zn	Ca_P_Mg	Ca_P_Sr	F	P
Pyruvate	5.32 ± 0.28	5.12 ± 0.19 (*)	5.05 + 0.15 (*)^a^	5.16 ± 0.24 (*)	5.25 ± 0.56^a^^,^^b^	5.22 ± 0.72^b^	20.48	<0.001
Lactic acid	6.47 ± 0.18	5.28 ± 0.82 (*)^a^	4.98 ± 0.23 (*)^a^	5.02 ± 0.84 (*)^a^	5.09 ± 0.41 (*)^a^	5.97 ± 0.17 (*)^b^^,^^c^^,^^d^^,^^e^	8.72	<0.001
fructose-6-phosphate	5.56 ± 0.14	5.42 ± 0.36	4.96 ± 0.17 (*)^a^	5.02 ± 0.21 (*)^a^	5.18 ± 0.31 (*)	3.49 ± 0.26 (*)^a^^,^^b^^,^^c^^,^^d^^,^^e^	12.54	<0.001
Cyclohexanecarboxylic acid	5.51 ± 1.32	5.55 ± 1.52	5.35 ± 1.91	5.57 ± 1.56	5.50 ± 0.98	5.53 ± 1.46	0.03	6.25
Aminoadipic acid	5.86 ± 0.92	5.92 ± 0.84	5.73 ± 0.72	5.86 ± 0.73	5.82 ± 0.37	5.88 ± 0.74	0.15	4.58
Citric acid	8.00 ± 0.85	7.53 ± 0.46 (*)	7.86 ± 0.56	6.80 ± 0.42 (*)^a^	7.47 ± 0.47 (*)	6.07 ± 0.62 (*)^a^^,^^b^^,^^c^	15.36	<0.001
Indole-3-acetic acid	4.28 ± 0.51	4.94 ± 0.28^a^	4.21 ± 0.81^b^	4.34 ± 0.54^b^	4.39 ± 0.19^b^	4.31 ± 0.43^b^	13.78	<0.001
Bovinocidin	4.31 ± 0.85	4.24 ± 0.68	4.00 ± 0.71 (*)	4.17 ± 1.8 (*)	4.21 ± 0.94	5.33 ± 0.67^a^^,^^b^^,^^c^^,^^d^^,^^e^	12.87	<0.001
Terephthalic acid	5.56 ± 0.54	5.60 ± 0.37	5.51 ± 0.46	5.75 ± 0.73	5.57 ± 0.82	5.58 ± 0.31	0.11	5.73

a Indicates *P* < 0.05 compared with the NC group.

b Indicates *P* < 0.05 compared with the Ca/P group.

c Indicates *P* < 0.05 compared with the Ca/P + NaF group.

d Indicates *P* < 0.05 compared with the Ca/P + Zn group.

e Indicates *P* < 0.05 compared with the Ca/P + Mg group.

**Figure 7:**
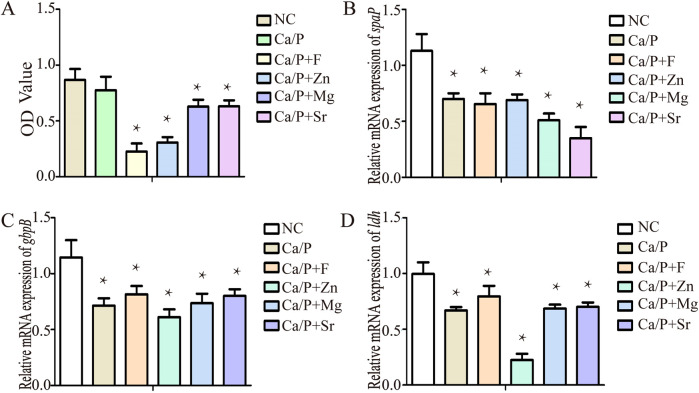
**(A)** Bacterial proliferation; **(B)** *spaP* gene expression; **(C)** *gbpB* gene expression; **(D)** *ldh* gene expression.

In Figure 7, Bacterial quantity, transcription of acid-producing genes, and adhesion genes all contribute to increased acidic metabolic products and tooth demineralization. The transcription levels of genes related to bacterial proliferation, adhesion, and acid production were evaluated. Bacterial proliferation was significantly inhibited in the experimental groups. The Ca/P + F group showed the strongest antibacterial effect, followed by the zinc group (Figure 7A). The differences were statistically significant; however, no significant difference was observed in the Ca/P group compared with the control group. The expression levels of bacterial adhesion-related genes, such as *spaP* and *gbpB*, decreased in the experimental groups. Among these, *spaP* expression decreased notably in the Sr group, and *gbpB* expression decreased significantly in the zinc group. All differences were statistically significant. The expression of the bacterial acid-production gene *ldh* also declined, with the most substantial reduction observed in the zinc group. Compared with the control group, the reductions in other groups were also statistically significant. Overall, the results suggest that the experimental group may reduce bacterial acidic metabolite production via modulation of the glycolytic pathway.

## 4 Discussion

Commonly used methods for preventing early dental caries, such as mineralizing solutions and fluoride, are widely applied in oral health care and yield favorable short-term results. However, robust evidence supporting their long-term efficacy is lacking (12). Long-term effectiveness largely depends on the interaction between cariostatic agents and the tooth surface, and different preventive approaches produce varying outcomes (13). Therefore, comparative studies are warranted. Calcium and phosphorus can be deposited onto tooth surfaces or demineralized teeth. Fluoride can form fluorapatite, which is more resistant to acid, with tooth enamel. Zinc reduces bacterial and tartar formation on tooth surfaces (9). Magnesium prevents biofilm formation, thereby decreasing the risk of dental caries (10). Enamel carries negative charges, attracting Sr ions to form a Stern layer. This layer improves the acid resistance of teeth, significantly preventing early dental caries (11). All these components have demonstrated caries-preventive properties. This study compared their effects on tooth acid resistance, bacterial adhesion, bacterial metabolic acid production, and their relative advantages and disadvantages in caries prevention.

The first step involves surface observation, elemental analysis, and identification of possible compounds formed. Teeth were immersed in different mineralization solutions. SEM revealed the smoothest deposits in the Mg group, whereas zinc and Sr groups formed granular structures (Figure 1). Energy spectroscopy results indicated that fluoride showed the lowest deposition rate, Strontium occurs in only trace amounts in natural teeth and is often undetectable. While zinc most readily deposited onto the tooth surface (Table 4). Zinc exhibits the highest affinity for binding to the tooth surface. Although all tested substances can deposit on the enamel surface, whether they form chemical bonds remains uncertain. Substances formed on the surfaces were further analyzed. Based on XRD and XPS results, fluorapatite was confirmed to form from the reaction of fluoride with hydroxyapatite (Figures 2A,E). Enamel consists of hydroxyapatite and organic substances. Organic substances also contain hydroxyl groups; however, no reaction was observed between fluoride and these hydroxyl groups to form new compounds. No precipitation of calcium fluoride was observed. Magnesium reacted with hydroxyl groups in hydroxyapatite, forming magnesium hydroxide (Figures 2C,H). XPS peaks of zinc, Sr, and magnesium suggested formation of metal salt compounds, such as sulfates, carbonates, and phosphates. However, no definitive crystalline forms were detected by XRD. Infrared spectroscopy further confirmed the presence of phosphate and carbonate ions (Figure 2I). Therefore, these cations were inferred to possibly form phosphates with hydroxyapatite or carbonates with organic matter. Further analysis is required to determine the exact molecular formulas. Nevertheless, these results confirm that all substances react with tooth surfaces. Comparative analysis suggests that fluoride and magnesium reliably form new compounds with the tooth surface, enabling chemical bonding and stronger adhesion.

The next step is to assess physical and chemical properties, including post-treatment acid resistance and changes in frictional characteristics. Acid etching was performed on the teeth after treatment. All treated teeth demonstrated improved acid resistance on their surfaces (Figures 3A–C), consistent with previous studies (9–11). The fluorine, zinc, magnesium, and Sr groups showed greater acid resistance than the blank and calcium-phosphorus groups, with fluorine exhibiting the most notable improvement. Moreover, surfaces of the fluorine, zinc, and magnesium groups remained relatively uniform after acid etching (Figure 3C). Wear resistance of teeth was evaluated using friction tests (Figure 3D). The three-dimensional images indicated that zinc, magnesium, and Sr groups had lower wear resistance than the blank, calcium-phosphorus, and fluorine groups. Figures 3E–K support these findings, showing that the blank group had the highest wear resistance. Thus, these elements improve tooth acid resistance but may reduce surface wear resistance. Comparative evaluation of surface structures after acid erosion and abrasion testing revealed that fluoride most effectively enhances enamel acid resistance and exhibits the greatest wear resistance.

Furthermore, we examined the effects of treated teeth on bacterial adhesion, acid production, and metabolite profiles. Bacterial adhesion is essential for bacterial acid erosion. Proline-rich acidic proteins (PRPs) in human saliva adhere to tooth enamel and selectively adsorb bacteria (14). To better simulate clinical conditions, saliva was filtered to remove bacteria, teeth were soaked, and bacterial adhesion experiments were performed. SEM (Figures 4–7) revealed bacterial metabolites as the primary substances adhered to tooth surfaces; bacterial cells were only clearly visible in the Sr group. Fewer bacteria adhered in the blank group compared to the Sr group. Possible reasons include that under natural conditions, *S. mutans* forms plaque with multiple bacterial species, enhancing enamel adhesion (15). In this study, only *S. mutans* was used, potentially limiting bacterial adhesion. This analysis revealed that metabolic products predominantly adhered to the tooth surface, with relatively low bacterial attachment. To determine whether bacterial adhesion was higher in the strontium group compared with others. Hydrophilicity tests further showed that the Sr group exhibited significantly greater hydrophilicity compared to other groups (Figures 4G–M), explaining increased bacterial adhesion. Culture medium analysis revealed higher pH values in experimental groups than in the blank group (Table 5), confirming that these substances inhibit bacterial acid production. Fluorine showed the most significant inhibition of pH reduction. The zinc group also showed a significant inhibitory effect on pH reduction. Figure 4N presents a metabolite classification plot, in which greater inter-sample distances indicate greater differences in metabolite composition. Metabolite analysis demonstrated marked differences between the fluorine group and the other five groups (Figure 4N). We postulate that fluorine, the only anion among the tested elements, inherently differs in behavior from the metallic elements. The classification of these elements inherently involves significant differences.

Based on these findings, acidic metabolites in the experimental groups differed from those in the control group, prompting further investigation into potential signaling pathways underlying these changes.

To explore the reasons for metabolite changes, related signaling pathways were investigated using KEGG metabolic pathway analysis (Figure 5). The results showed that fluorine mainly influenced pathways related to antibiotic and alkaloid biosynthesis in *S. mutans*. The Ca/P group primarily affected lipopolysaccharide and various amino acid synthesis pathways. Zinc mainly influenced amino acid and coenzyme biosynthesis pathways. Magnesium mainly affected lipopolysaccharide and steroid synthesis pathways, while Sr predominantly impacted citric acid and alkaloid synthesis pathways. It is hypothesized that strontium and fluorine may elevate pH by forming basic compounds, while zinc and magnesium mainly influence bacterial proliferation. Magnesium may also modulate bacterial adhesion strength. Based on these results, metabolites influencing acid-base balance were analyzed (Figure 6). Acidic amino acids such as creatine, glutamic acid, and galactonic acid were prominently synthesized in the blank group, whereas their synthesis decreased most significantly in the fluorine group, followed by magnesium, zinc, Sr, and Ca/P groups, respectively. These organic acids likely play a role in the bacterial glycolysis pathway.

Finally, based on the observed changes in organic acids, we hypothesize that these anti-caries elements may affect the acid production of *S. mutans* through glycolysis-related pathways. Sodium fluoride inhibits glycolytic ATP synthesis (16). Zinc inhibits certain dehydrogenases; specifically, by interfering with *ldh* activity, zinc hinders the conversion of pyruvate to lactic acid. In glycolysis, magnesium binds phosphorylated substrates, facilitating phosphate transfer via phosphokinase enzymes. However, high magnesium concentrations can inhibit this reaction (17). Current studies have not clarified the relationship between Sr and glycolysis. Table 6 indicates that the experimental groups produced fewer organic acids compared with the blank group, with the most significant reductions observed in the zinc and fluorine groups. This finding is consistent with the measured pH results. Bacterial proliferation analysis revealed the strongest antibacterial effect with fluorine, followed by zinc. Previous research showed significant inhibition of *S. mutans* proliferation by NaF at concentrations of 1 mM and 2 mM (18), supporting our findings. Referring to KEGG enrichment pathway data, this antibacterial effect might be related to alterations in the antibiotic biosynthesis pathways of *S. mutans*. Fluorine is thought to inhibit phosphoenolpyruvate formation, reduce glucose transport and metabolism, and interfere with glycolysis. Zinc disrupts *S. mutans* cell membrane integrity, inhibiting bacterial proliferation (19). Magnesium primarily reduces bacterial adhesion in the oral cavity and alters bacterial morphology (20). Sr-doped cement reduces adhesion and proliferation of cariogenic bacteria (21). These substances have been demonstrated to exert antibacterial effects via different mechanisms. These mechanisms may require further experimental verification. RT-PCR was conducted to validate key glycolysis and bacterial adhesion genes. Expressions of adhesion-related genes (*spaP* and *gbpB*) were reduced in experimental groups. *SpaP* gene expression significantly decreased in the Sr group, contradicting the adhesion experiment results. This discrepancy might be due to the weak antibacterial effect of Sr, leading to increased bacterial populations and enhanced adhesion. Firstly, we suggest that the antibacterial effect of strontium is relatively weak, resulting in higher bacterial counts in the culture medium (Figure 7A) and increased bacterial adhesion. Additionally, the expression of *gbpB* in the strontium group was higher than in other groups, potentially because strontium promotes *gbpB* transcription, thereby enhancing bacterial adhesion, rather than affecting *spaP.* The adhesion mechanism of *spaP* primarily involves strengthening bacterial binding to type I collagen on the tooth surface. Although this binding is strong, it requires a longer time (approximately 20 cycles) to establish (22). Thus, low *spaP* transcription in the strontium group may lead to less firm bacterial adhesion without reducing the total number of adherent bacteria. Finally, the strontium group exhibited the highest hydrophilicity, which may further facilitate bacterial adhesion. *GbpB* gene expression significantly decreased in the zinc group, possibly due to effects on lipopolysaccharide pathways identified by KEGG analysis. *Ldh*, crucial for bacterial glycolysis, directly influences acid production by *S. mutans*. *Ldh* gene expression significantly decreased in the experimental groups, with the greatest decrease observed in the zinc group. Fluorine, while the most commonly used anti-caries agent, carries certain risks and can even lead to major public health concerns. Some scholars have questioned whether fluoride should be added to drinking water (23). Findings from this study indicate that zinc exhibits a significant antibacterial effect and effectively prevents pH reduction, suggesting its potential as an alternative to fluoride.

In recent years, the application of incorporating elements such as fluorine, zinc, magnesium, and strontium into oral materials for caries prevention has gradually emerged as a new research trend. The present study explored the various caries-preventive properties of these elements when used as mineralizing solutions, and the relevant findings can lay a foundation for subsequent clinical trials. A notable limitation of this study is that we did not conduct transcriptomic or proteomic analyses, nor did we explore the mechanisms behind metabolite differences and pH changes in greater depth, so we plan to investigate these aspects more thoroughly in subsequent experiments. Additionally, this study used extracted teeth, which may differ from the *in vivo* state, and to better align with clinical scenarios, we will also perform more in-depth experiments in future research.

## 5 Summary

Among reactions on the tooth surface, Mg had the least impact, resulting in the smoothest surface. Zinc was most readily deposited, whereas Sr had the lowest surface deposition. Fluorine formed fluorapatite, but did not significantly react with enamel proteins nor form substantial calcium fluoride. Magnesium formed magnesium hydroxide, while zinc, magnesium, and Sr combined with phosphates or carbonates. All these substances enhanced tooth acid resistance, with fluoride showing the greatest effect and highest friction resistance. Using *S. mutans* as a bacterial model, bacteria showed limited adhesion to tooth surfaces, with metabolites primarily adhering instead. The Sr-treated surface exhibited the highest bacterial adhesion and best hydrophilicity. Fluorine significantly altered bacterial metabolites, differing markedly from other groups. Fluorine also most effectively inhibited acid production by *S. mutans*, followed by zinc. Metabolic pathway analysis revealed fluorine primarily affected antibiotic and alkaloid biosynthesis, whereas Ca/P, zinc, and magnesium mainly influenced lipopolysaccharide, steroid, and citric acid synthesis pathways. Fluorine most strongly inhibited acidic amino acid and organic acid formation, and also demonstrated the strongest antibacterial activity, followed by zinc. However, zinc had a stronger inhibitory effect on *ldh* transcription compared with fluorine. Sr had the strongest inhibitory effect on *spaP* adhesion-factor transcription.

Since fluoride is both the most commonly used and the most hazardous anti-caries agent, these findings highlight the significant antibacterial activity of zinc and its ability to prevent pH reduction. Zinc can also enhance tooth resistance to acid. In clinical practice, it is worth investigating whether zinc could replace fluoride. The antibacterial efficacy, abrasion resistance, and solubility of fluoride, zinc, strontium, magnesium, and calcium are all lower than those of tooth enamel. This suggests that their clinical application should be limited to caries-prone areas, such as fissures or proximal surfaces, which are less susceptible to wear. All concentrations used in this study were saturated solutions. For clinical use, rubber dam isolation is necessary. Currently, anti-caries agents are typically applied in the form of foams or gels with sustained-release properties. Our future research will replicate these clinical formulations and develop zinc-based gels or foams for application.

## Data Availability

The original contributions presented in the study are included in the article/Supplementary Material, further inquiries can be directed to the corresponding author/s.
